# Computer Vision for Continuous Bedside Pharmacological Data Extraction: A Novel Application of Artificial Intelligence for Clinical Data Recording and Biomedical Research

**DOI:** 10.3389/fdata.2021.689358

**Published:** 2021-08-27

**Authors:** Logan Froese, Joshua Dian, Carleen Batson, Alwyn Gomez, Amanjyot Singh Sainbhi, Bertram Unger, Frederick A. Zeiler

**Affiliations:** ^1^ Biomedical Engineering, Faculty of Engineering, University of Manitoba, Winnipeg, MB, Canada; ^2^ Section of Neurosurgery, Department of Surgery, Rady Faculty of Health Sciences, University of Manitoba, Winnipeg, MB, Canada; ^3^ Department of Anatomy and Cell Science, Rady Faculty of Health Sciences, University of Manitoba, Winnipeg, MB, Canada; ^4^ Section of Critical Care, Department of Medicine, Rady Faculty of Health Sciences, University of Manitoba, Winnipeg, MB, Canada; ^5^ Centre on Aging, University of Manitoba, Winnipeg, MB, Canada; ^6^ Division of Anaesthesia, Department of Medicine, Addenbrooke’s Hospital, University of Cambridge, Cambridge, United Kingdom

**Keywords:** computer vision, image modification, opitcal character recognition, system integration, data integration

## Abstract

**Introduction:** As real time data processing is integrated with medical care for traumatic brain injury (TBI) patients, there is a requirement for devices to have digital output. However, there are still many devices that fail to have the required hardware to export real time data into an acceptable digital format or in a continuously updating manner. This is particularly the case for many intravenous pumps and older technological systems. Such accurate and digital real time data integration within TBI care and other fields is critical as we move towards digitizing healthcare information and integrating clinical data streams to improve bedside care. We propose to address this gap in technology by building a system that employs Optical Character Recognition through computer vision, using real time images from a pump monitor to extract the desired real time information.

**Methods:** Using freely available software and readily available technology, we built a script that extracts real time images from a medication pump and then processes them using Optical Character Recognition to create digital text from the image. This text was then transferred to an ICM + real-time monitoring software in parallel with other retrieved physiological data.

**Results:** The prototype that was built works effectively for our device, with source code openly available to interested end-users. However, future work is required for a more universal application of such a system.

**Conclusion:** Advances here can improve medical information collection in the clinical environment, eliminating human error with bedside charting, and aid in data integration for biomedical research where many complex data sets can be seamlessly integrated digitally. Our design demonstrates a simple adaptation of current technology to help with this integration.

## Introduction

Current therapeutic interventions in Traumatic Brain Injury (TBI) are generally based on low frequency physiological response over large sample sizes, focusing on long epoch outcomes (Chalmers et al., 1981; Carney et al., 2017). Though this methodology can be effective in identifying large global phenomenon, momentary individualized events are masked within these large datasets. Thus, methodologies are emerging that leverage higher frequency data to find momentary phenomenon that focus on individualized patient response to medical treatment (Carney et al., 2017; Matchett et al., 2017; Zeiler et al., 2018a). Furthermore, within TBI care, recent literature has emerged connecting high frequency physiology with TBI outcome (Balestreri et al., 2015; Cabella et al., 2017; Zeiler et al., 2018b). Yet, few studies connect the momentary response of high frequency physiology to current hourly recorded therapeutic infusions (Froese et al., 2020a; Froese et al., 2020b; Klein et al., 2020). Through the use of more robust and individualized datasets, treatment guidelines can be focused on patient specific healthcare interventions which can lead to more individualized and personalized care. To take advantage of emerging technologies and new health metrics, real time high frequency physiological and treatment care data needs to be recorded and integrated. However, despite this increase in computational integration within health care, there are countless devices that are either released with insufficient digital output or are simply too outdated to carry the necessary hardware infrastructure to output the required data at a high frequency. This is particularly the case with many commercially available and clinically utilized medication pumps. As such, treatment information in many instances is still recorded manually at low frequency in bedside charts, or e-charts. Such methods are prone to errors in data entry and are time consuming for clinical staff.

The limited compatibility of many bedside medical devices hinders clinicians’ ability to capture high frequency data, thus there is a need to leverage interfaces that convert such data from bedside devices directly into digital data. Many medical devices use text displays to convey the required information to the user. The text display therefore has the desired information, but based on the antiquated hardware, it lacks the compatibility to convert the information to a digital format. This problem is described as Text Information Extraction (TIE) (Jung et al., 2004) and has been addressed in other environments like text-based image processing, (Park et al., 1999; Kim et al., 2002; Carvalho, 2016) document decoding (Cheng et al., 1997; Feng et al., 2006) and video text extraction (Locating Characters in Sc, 1047; Fischer et al., 1995). All of these systems extract alphanumeric characters using Optical Character Recognition (OCR) via computer vision techniques, which leverage artificial intelligence to convert image characters into digital data (Schantz, 1982). This method, although well documented, has yet to be adapted for the use and conversion of medical monitoring equipment. Therefore, with the emergence of new openly available software and the universal nature of personal computers, there is a potential to adapt past medical devices to the computational age.

Furthermore, for the integration of many older medical devices the only feasible solution to digital integration is through the use of scripting (Carvalho, 2013; Delaney et al., 2013; Carvalho, 2021). Likewise, as clinical data collection exceeds the limits of humans, the need to leverage scripting to ensure accurate data collection becomes necessary (Mardis, 2011; Delaney et al., 2013). To bridge this gap in compatibility, we have endeavoured to build a system that uses a camera to attain real time output from a text based display screen from bedside intravenous medication pumps and convert it into a continuously updated digital format to be captured and linked with other time-series data at the bedside in real time.

## Materials and Methods

### Device Set-Up and Image Capture

**FIGURE 1:**
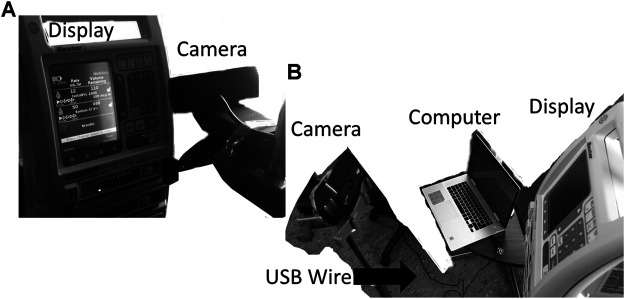
Setup for the camera and pump. General setup for our design, with the monitor display being captured through an external camera is displayed in image **(A)** and **(B)**. In figure **B** the USB wire connecting the computer to camera can be seen. The current design has the camera directly in front of the text display.

**FIGURE 2:**
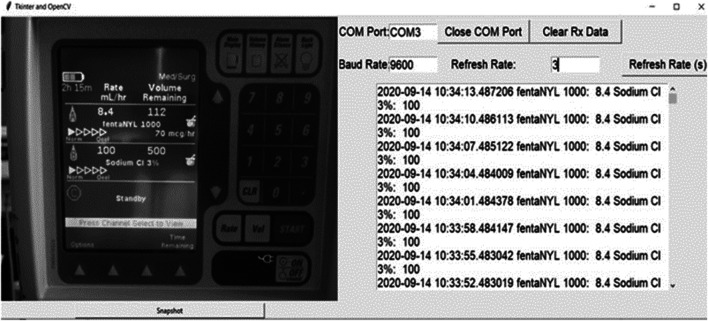
Python interface. **Left Panel**–Displays digital photo of medication pump taken by the camera, **Right Panel** - Displays the interface of our system, with real time data being updated from the extracted features from the medication pump display.

This work was conducted at the Winnipeg Acute TBI Laboratories, at the University of Manitoba. The set-up consisted of a USB connected camera (Logitech C920s Pro HD Webcam, Logitech, Newark, CA, United States) to take real time images of a commercially and commonly available intravenous medication pump (Baxter Colleague 3 CXE, Baxter Canada, Mississauga, Canada) which currently has no digital outport. Images are captured at 60 frames/second from a USB camera and copied directly onto a basic consumer laptop, see Figure 1. The full Python scripting language code (Python 3, Scotts Valley, CA: CreateSpace) can be found in either Supplementary Appendix A or GitHub (https://github.com/lofro/TIE_OCR). The basic operation of this system leverages 4 main libraries in python; “pytesseract,” “cv2,” “serial” and “tkinter.” “pytesseract” and is used for the OCR processing. (Lee, 2007) “Cv2” is also an image processing and manipulation library. (Bradski, 2000) The use of these libraries will be detailed in the subsections to follow. “Serial” is a library in python that allows for the creation and use of serial sockets (Welcome to PySerial’s Documentation PySerial 3.4 Documentation). Finally, we used the “tkinter” library to create the display and user interface that is seen in Figure 2 (Lundh, 1999). To create a video we leveraged the “cv2. CaptureVideo” function to extract frames and the “tkinter.Canvas” to display these frames. When either the *snapshot* button is press or the time delay is reached, the current frame captured will be processed.

### Image Processing and Feature Extraction

**FIGURE 3:**
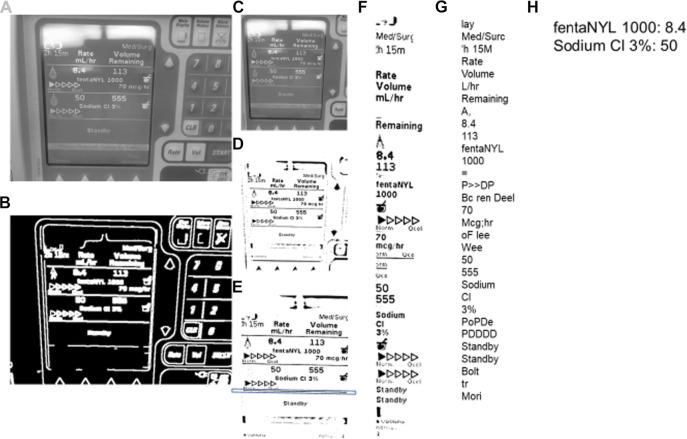
Steps for image processing–TIE and OCR. TIE = Text Information Extraction, OCR = Optical Character Recognition. The processing proceeds in alphabetical order. **(A)** is the initial image converted to grayscale. **(B)** is the grayscaled image processed with the Canny function. **(C)** is the grayscaled cropped image using the rectangle contours of image **(B)**. **(D)** is the adaptive mean threshold function of image **(C)**. E is the cropped and rotated image **(D)** with the key horizontal line contained within a box. F is the features found with the google tesseract of image **(E)**. **(G)** is the string that Google Tesseract output from image **(F)**. **(H)** is the final output of the **(G)** string process.

The TIE for these images was performed using Python. On the initiation of the code, an interface for the image capture will appear, as shown in Figure 2. The subsequent image manipulations are demonstrated in Figure 3, which illustrates our method to solve the TIE problem. The TIE problem can be divided into the following sub-problems: detection, localization, tracking, extraction/enhancement, and recognition (Jung et al., 2004). Within our design we focused on localization, extraction/enhancement and recognition, as we can assume the images captured have some form of desired information, and that the features of interest stay relatively constant.

An image can be captured manually or automatically after an allotted time. Once the image is captured it goes through the entire TIE image processing as seen in Figure 3, proceeding alphabetically going from A to H. The two TIE subgroups of image localization and extraction/enhancement are performed in unison, shown in Figures 3A–E. Initially the image is converted to grayscale using a “cv2” function (Figure 3A), then using Canny edge detection, the image edges are traced (Figure 3B). (Open(X). Canny Edge) Canny highlights the edges of an image using the intensity gradient of the image, which is the color difference on local pixels to find the edge of shapes within the image (Canny, 1986). Using these edges, we can differentiate the display from the larger image by the rectangular aspect of the display. To do this the edges are grouped into contours. Contours are the bounding points that map the outline of a continuous white shape of Figure 3B. Each continuous white shape is bounded by the smallest, best fitting rectangle that contains all the contours of that group. With all shapes having a respective bounding rectangle, the largest area rectangle can be found, which is assumed to be the display screen and used to give Figure 3C.

The image is then enlarged to improve the small feature edges for the adaptive mean threshold. The adaptive mean threshold uses the area of local pixel brightness to find a mean brightness which then can be contrasted against the pixel of interest to identify if it should be black or white, resulting in Figure 3D. (Open(X). Image Thres) Next, the contours of Figure 3D are found in a similar way as before, (using canny edge detection) with the exception that it looks for the continuous black portions. Like before, the continuous black shapes are all bounded by a rectangle and used with their respective contours to rotate the image and crop the image for a second time. To rotate the image, a key horizontal line is needed (highlighted by the box around a line in Figure 3E), this line is found by using the relative height to length of the bounding rectangle. The bounding rectangle must have a width greater than ¾ of the image width, and of the rectangles that meet this criterion, the one with the smallest height is chosen. Next, with the contours from which the previously described bounding rectangle encompasses, the line of best fit is made. That being, a best fit line is drawn through the key horizontal line. This is the least squares regression line with the contours as the points of interest. The best fit line is created using a “cv2” function and has an output of a location and an angle of rotation. (Bradski, 2000) This angle of rotation is also the angle for the image to rotate. To find the cropping area, the width and location of the bounding rectangle for the key horizontal line is used to find the x component of the cropped image (the horizontal location and width). The y component (vertical location and height) is assumed to be at the 5 and 90% of the initial image height, which allows the image to be cropped (Figure 3E). This concludes the localization of the TIE process as the image is focused on only the text display. The last step in enhancement/extraction is performed using Google Tesseract’s (Google Inc., https://github.com/tesseract-ocr/tesseract/) feature selection function, this function uses an artificial intelligence algorithm to find all key shapes within the image. (Lee, 2007) These are then cropped from the initial image and displayed in a consecutive order to give Figure 3F.

### Character Recognition

**FIGURE 4:**
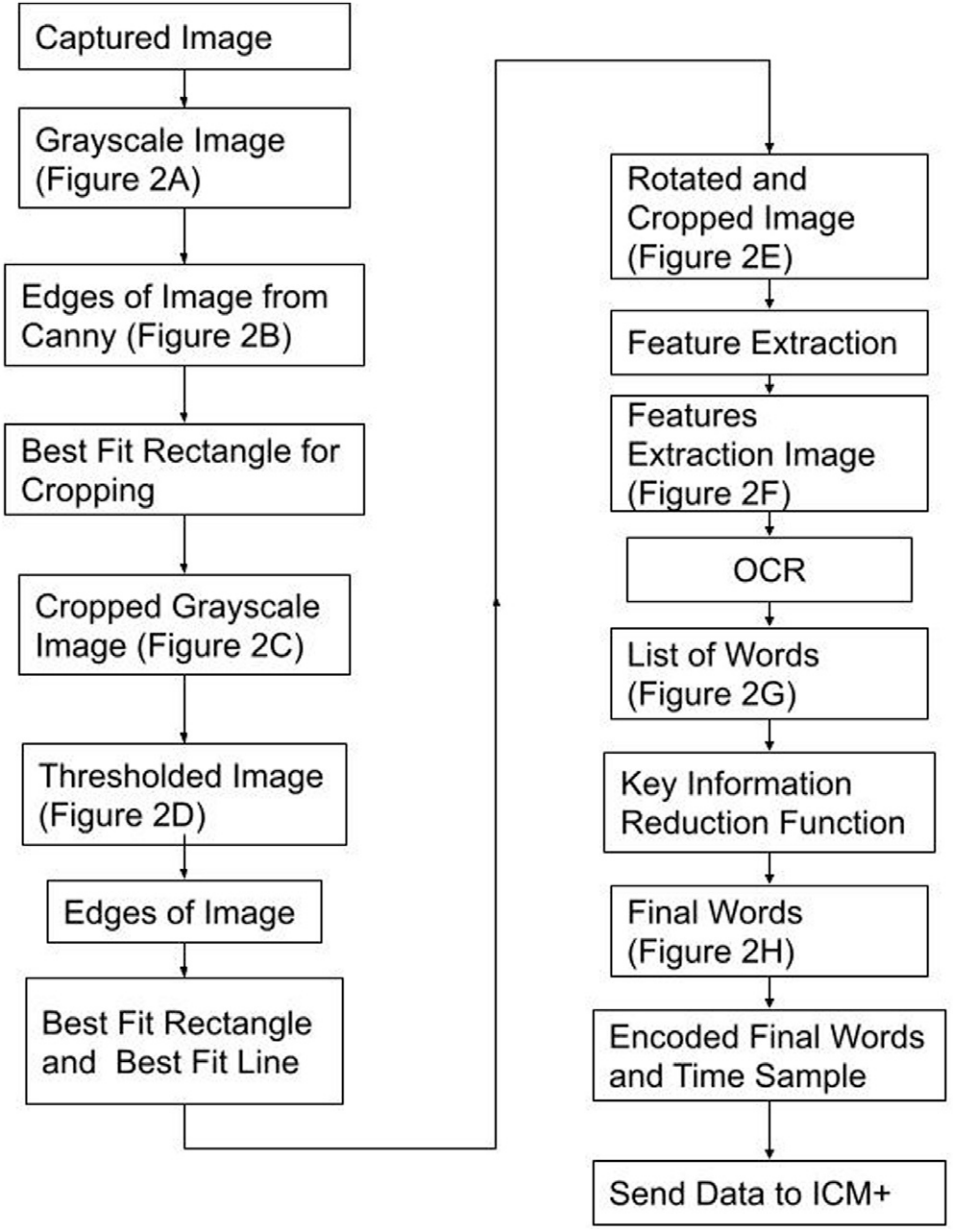
Process Map–From Image Capture to Serial Output Figure displays the process taken to convert the image into its digital information and the steps to send the data to ICM+ (data acquisition platform). The best fit rectangles and line are the key shapes used to crop and rotate the image. Feature Extraction is a Google Tesseract function to find key shapes. Key Information Reduction Function is a function used to find and order the alphanumeric of interest.

The last part of the TIE process, recognition, uses Google Tesseract OCR (Lee, 2007) to give the output text shown in Figure 3G. This process, like all OCR, involves comparing a library of identified shapes to the data, in this way the best matched letter is assumed. (Lee, 2007) From Figure 3G the desired values are extracted based on the nature of the OCR output and design of the text display, that being, the dose is always followed by the dose amount and left/time remaining, and the medication type is found by a list of predefined words of interest. Together the dose amount and medication can be paired up, and in almost any fashion given as Figure 3H. To improve accuracy, we found the key words (those being greater then 4 characters of alphabetical values) and connected those with a number in a similar location, for the full OCR code see Supplementary Appendix A.2. From here the data is digitized and can be output into any desired format. A full process map of the above TIE and OCR processes, from image capture to serial output can be seen in Figure 4.

### Digitized Data Capture

Using a virtual serial port, we sent the serialized data (Figure 3H) to Intensive Care Monitoring “Plus” (ICM+) (Cambridge Enterprise Ltd., Cambridge, United Kingdom, http://icmplus.neurosurg.cam.ac.uk), generating continuously updating real time data (Figure 2). The virtual serial port is an internal design that acts like serial port for any RS232 ASCII streaming device and was made using freely available software (null-modem emulator (com0com), http://com0com.sourceforge.net). (Hatchett, 1991) In ICM+ the data was parsed into the desired functions identical to the parsing of any other device data. ICM+ was used as an example of a data acquisition platform for the continuous time-series capture of such data, as it is the platform utilized by our laboratory for bedside physiology research. The above-described design can be integrated with any data acquisition platform which can record serial data.

**FIGURE 5:**
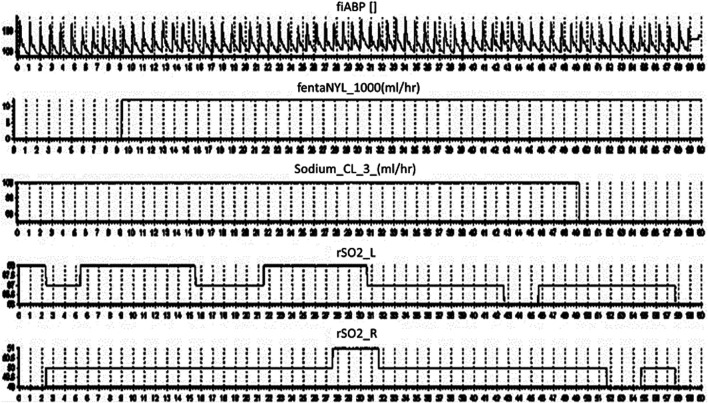
ICM + Final Output. Displays the final output on ICM + over a 60 s period from top to bottom; the arterial blood pressure, fentanyl, sodium chloride and regional oxygen saturations.

Finally, to show-case the capture of continuous medical pump data in conjunction with other monitoring devices, we recorded continuous bifrontal cerebral regional oxygen saturations using near infrared spectroscopy (Covidien INVOS 7100, Medtronic Canada) and continuous non-invasive arterial blood pressure through a finger-cuff technique (Finapres NOVA Nanocare, Finapres Medical Systems, Enschede, Netherlands, http://www.finapres.com/home), in a volunteer. The regional oxygen saturation was sampled at 1 Hz, while the arterial blood pressure was sampled at 250 Hz. Therefore, we can run our system in parallel with any number of compatible devises as can be seen in Figure 5.

## Results and Discussion

### System Performance

As this entire system was a proof of concept, the design proves that there is technology available to complete an effective TIE process on a human-based text interface output, using an intravenous medication pump as an exemplar (examples of captured frames that worked can be seen in Supplementary Appendix B). Furthermore, the design used only a common camera, a laptop and freely available open source software, (Lee, 2007; Bradski, 2000; Hatchett, 1991) demonstrating the accessibility of this conversion system.

Though we built a working prototype, there were some key issues that arose when operating the system. The first and perhaps most important, is the slightly inconsistent nature of the OCR recognition which has been documented in the past (Carvalho, 2016; Schantz, 1982; Lee, 2007). When implementing OCR, there is a tendency for letters and word orders to be mismatched. For example, a common error is the letter “f” interpreted as a “t,” i.e. “tentanyl” instead of “fentanyl.” This can be bypassed by backend language algorithms and deep learning techniques (Mokhtar et al., 2018; Le et al., 2019). Another common issue encountered is the mismatch of numbers “5,” “6”, “8” and “9,” which in operation have become interchangeable with one another if the image is insufficiently processed. To overcome this problem in operation, converting the image to Figure 3F, with significant space between the lines of text, improved recognition. Also, the enlargement of features made the edges more robust (improving extraction/enhancement of the image). Though it must be acknowledged, in our described design and camera setup, we did not require these improvements to get sufficiently accurate data. Such modifications may be necessary with cheaper and lower resolution cameras.

The second issue is the interference that background noise can have on the image, which interferes with extraction and enhancement. If the display is dim, with a light that reflects directly in the camera, there are scenarios in which the captured image data can be masked behind this light. Likewise, if the camera is moved into such an angle as to obscure the image, the OCR software fails to accurately extract the information. Currently, there are no working examples that we know of that effectively adjust images at obscure angles to effectively output a coherent final image however, there are emerging proposed solutions (Oakley and Satherley, 1998; Li and Doermann, 1999; Li et al., 2000; Witten et al., 2004). Therefore, in the implementation of this design the most effective solution is setting up the camera to extract clear, centered images.

### Reflections on Impact of the Designed System

#### The TIE/OCR Process

This system of converting real time data from a medical device display into digital data, is the first that we have knowledge of. As such, this system illustrates that there is a bridge between computers and older devices that lack the necessary compatibility, using TIE processing. In this way there is an opportunity to extract data even when there is no capability of directly accessing the digital port, or when no digital output is offered. However, the design and operation of this system enforces the desire for a robust TIE methodology, due to the tenuous precision in the output. The mixed precision is caused by errors mostly relating to the OCR methodology for recognition, thus the field of text extraction is expanding with new developments and emerging improvements to all aspects of the TIE processing. These include word detection using Markov Random Field (Yalniz and Manmatha, 2019) and canonical correlation analysis, enhancing image quality by layering multiple images, (Wemhoener et al., 2013) smoothing edges by using corner detection, (Yalniz and Manmatha, 2012) and having more robust feature detection methods (Witten et al., 2004; Oakley and Satherley, 1998; Li et al., 2000; Li and Doermann, 1999) with more areas and designs proposed to improve information retrieval from images (Allan et al., 2003). These improvements highlight ideas to incrementally change the TIE methodology and enhance text extraction. Furthermore, by leveraging Deep Learning techniques before and after the OCR process, the shortcomings that are inherit with the OCR could be addressed. The two key areas to apply these Deep Learning solutions would be the creation of the improved text images (Figures 3E,F) and error correction (Figures 3G,H), which have emerging methods to address them (Mokhtar et al., 2018; Le et al., 2019; Namysl and Konya, 2019; Yin et al., 2019; Karthikeyan et al., 2021).

For individuals who endeavour to build a similar TIE system, the use of a prebuilt OCR is recommended. The open-source nature of Google Tesseract OCR makes it easily adaptable but supported under the Google banner also gives it access to a vast database to build its character recognition library on. As well, Google Tesseract OCR offers language conversion for over 50 different languages. (Lee, 2007) As global health becomes integrated, systems that can be adapted for a global community become imperative. These platforms bear the added benefit of being supported by a wide group of people, improving not only its functionality but its robustness as it pertains to various aspects including varying text font styles and languages. Therefore, although in theory it is possible to build one’s own OCR system, there is limited practical reason to do so.

#### Application to Bedside Medical Big Data

Aside from the novel application of computer vision to solve a digitization problem for medical device data, the TIE also offers the removal of the human element within data collection, as humans account for a large amount of the inconsistency within data processing (Barchard and Pace, 2011). In both the clinical care provision and biomedical research fields, data accuracy is critical. Errors in bedside or e-chart data entry, associated with human-based methods, can impact care delivery and safety for patients by allowing for treatment decisions to be made on inaccurate information. Similarly, accuracy of data in biomedical research is paramount as the focus of care becomes more responsive and individualized.

The TIE also improves the volume and frequency of data collection from such medical devices, exponentially higher than any human-based recording method. In almost all clinical data extraction, but in particular TBI data, the treatment methodologies are often updated at an hourly rate, with limited concern for the minute-to-minute fluctuations within care. Emerging studies in TBI research identify an optimal cerebral perfusion pressure which is coupled to minute to minute changes in physiology, (Steiner et al., 2002; Aries et al., 2012), with measures like intracranial pressure being well documented as having targeted goals to achieve (Cabella et al., 2017; Carney et al., 2017; Zeiler et al., 2020). Such targets require the implementation of high frequency data analysis, however the treatments associated with these goals is either undocumented, or lack precision in documentation as to the exact momentary changes within care. Thus, methods to improve time resolution, allowing data to be linked with other physiologic information for a clearer picture of treatment response/effect, is required, as highlighted in our example in Figure 5. Moreover, the nature of digitized information makes the update, dissemination, and archiving to prevent data loss a nearly trivial task. Thus, the breakdown or damage to one device can be mitigated by having continuous multi-connected data streams, limiting data loss.

## Future Directions

Despite the novel and interesting results described, future work is required in this area for further optimization. For this type of design there is a need to focus on three basic future implementations: the first, is creating a more robust TIE process with a focus on image enhancement and recognition. Such work will encompass variation in camera face angles and screen brightness/hues. The goal is to improve the efficiency of the output to more suitably honed results. Thus, the implementation of some previously proposed solutions to the OCR process using Deep Learning methods will be explored, including; convolutional neural networks, (Allan et al., 2003) neural machine translation techniques (Mokhtar et al., 2018) and provide improved lexicons.

The second area to address is a refined layout and interface. The goal for this style of technology is to have any user intuitively operate the device. As such, there will be work put in place to design a functional package that can be downloaded and will run like any other application. All of this will be done with freely available open-source software in order to promote the goal of improved data management and global health. One further aim is to expand applications to other medical devices and pumps that are commercially available.

Finally, to deploy this technology in both simulated and real-world healthcare environments. An example would be to setup this device in a simulation lab which is utilized to practice critical resuscitation skills for clinicians and trainees, prior to real-world application. Once feasibility and accuracy has been assessed in the simulated environment, the system can then be deployed in a real-world critical care environment here at the Health Sciences Centre in Winnipeg or other centers. Here real-time operational limitations will be explored, and the algorithms improved as needed. All future renditions and investigations will lead to improvements in the source code, which will be made openly available as new versions arise on GitHub.

## Data Availability

The raw data supporting the conclusion of this article will be made available by the authors, without undue reservation.
