# Supplementary material for: Computer Vision for Continuous Bedside Pharmacological Data Extraction: A Novel Application of Artificial Intelligence for Clinical Data Recording and Biomedical Research
Source: Front Big Data. 2021 Aug 27;4:689358. doi: 10.3389/fdata.2021.689358 (PMC8430398; doi:10.3389/fdata.2021.689358)
Supplement: Supplementary file 1 [file DataSheet2.docx]

Appendix B. Examples of Images that the System Extracts Desired Output


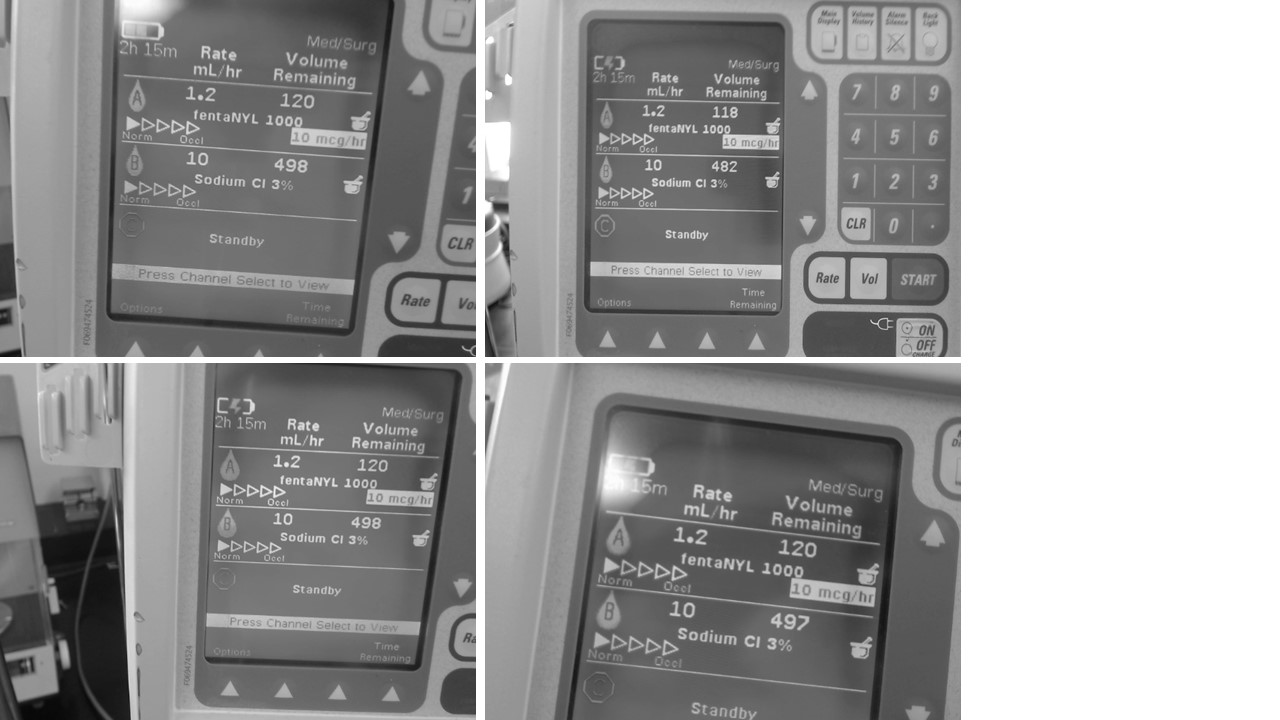


*Each of these four images were captured from the camera while running a real time serial output of the digitized data. The output was: “fentanyl 1000: 1.2 Sodium Cl 3%: 10” demonstrating the types of images that can be captured and still have a working system.*
