# Supplementary material for: Computer Vision for Continuous Bedside Pharmacological Data Extraction: A Novel Application of Artificial Intelligence for Clinical Data Recording and Biomedical Research
Source: Front Big Data. 2021 Aug 27;4:689358. doi: 10.3389/fdata.2021.689358 (PMC8430398; doi:10.3389/fdata.2021.689358)
Supplement: Supplementary file 2 [file DataSheet1.docx]

Appendix A. Python Code

The full working repository can be found at <https://github.com/lofro/TIE_OCR>. The code consists of three main files “Main.py”, “Img_to_Word.py”, “serial_rx_tx.py”. The Main.py creates the user interface and connect the user’s interactions to other systems. Img_to_Word.py converts a collected frame into an output of a desired string. Serial_rx_tx.py creates a serial port in python, it also handles the communication on the back end between the python interface and the serial output.

Appendix A.1. Main.py

import cv2

import tkinter #python quick user interface

import tkinter.scrolledtext as tkscrolledtext

import PIL.Image, PIL.ImageTk

import time

import sys

import datetime

import threading

import queue

#My libs

import serial_rx_tx

import Img_to_Word

#Initialize serial out port (will be COM3 at 9600 but can be changed)

serialPort = serial_rx_tx.SerialPort()

#Initialize log file

logFile = None

#Main app class

class App:

    # Inital code steps up all the user interface

    def __init__(self, window, window_title, video_source=0):

        self.thread_queue = queue.Queue() #queue for thread

        self.window = window # tkinter

        self.window.title(window_title)

        self.video_source = video_source

        self.outtext="Start"

        # open video source (by default this will try to open the computer webcam)

        self.vid = MyVideoCapture(self.video_source)

        # Create a canvas that can fit the above video source size

        self.canvas = tkinter.Canvas(window, width = self.vid.width, height = self.vid.height)

        self.canvas.grid(row=0,column=0, rowspan=10)

        # Comport

        self.label_comport = tkinter.Label(window,width=8,height=2,text="COM Port:")

        self.label_comport.grid(column=1, row=0)

        self.label_comport.config(font="bold")

        #COM Port entry box

        self.comport_edit = tkinter.Entry(window,width=8)

        self.comport_edit.grid(column=2, row=0)

        self.comport_edit.config(font="bold")

        self.comport_edit.insert(tkinter.END,"COM3")

        # COM Port open/close button

        self.button_openclose = tkinter.Button(window,text="Open COM Port",width=15,command=self.OpenCommand)

        self.button_openclose.config(font="bold")

        self.button_openclose.grid(column=3, row=0)

        #Clear Rx Data button

        self.button_cleardata = tkinter.Button(window,text="Clear Rx Data",width=15,command=self.ClearDataCommand)

        self.button_cleardata.config(font="bold")

        self.button_cleardata.grid(column=4, row=0)

        #Baud Rate label

        self.label_baud = tkinter.Label(window,width=8,height=2,text="Baud Rate:")

        self.label_baud.grid(column=1, row=1)

        self.label_baud.config(font="bold")

        #Baud Rate entry box

        self.baudrate_edit = tkinter.Entry(window,width=8)

        self.baudrate_edit.grid(column=2, row=1)

        self.baudrate_edit.config(font="bold")

        self.baudrate_edit.insert(tkinter.END,"9600")

        #Refresh label

        self.label_refresh = tkinter.Label(window,width=15,height=2,text="Refresh Rate:")

        self.label_refresh.grid(column=3, row=1)

        self.label_refresh.config(font="bold")

        #Picture refresh button

        self.button_senddata = tkinter.Button(window,text="Refresh Rate (s)",width=15,command=self.Refresh_Rate)

        self.button_senddata.config(font="bold")

        self.button_senddata.grid(column=5, row=1)

        #Refresh Rate entry box

        self.senddata_edit = tkinter.Entry(window,width=8)

        self.senddata_edit.grid(column=4,row=1)

        self.senddata_edit.config(font="bold")

        self.senddata_edit.insert(tkinter.END,"10")

        self.refresh_r=10

        # Textbox

        self.textbox=tkscrolledtext.ScrolledText(master=window, wrap='word', width=50, height=15) #width=characters, height=lines

        self.textbox.grid(column=1, row=3,columnspan=5)

        self.textbox.config(font="bold")

        # Button that lets the user take a snapshot

        self.btn_snapshot=tkinter.Button(window, text="Snapshot", width=50, command=self.snapshot)

        self.btn_snapshot.grid(column=0,row=10)

        ##### start multithread

        self.new_thread1 = threading.Timer(self.refresh_r,

            function=self.pic_to_text,

            kwargs={})

        self.new_thread1.daemon=True

        self.new_thread1.start()

        self.window.after(200, self.update_Image)

############### Take the image and convert it to Text and Send

    def pic_to_text(self):

        #Add new thread to multithread

        self.new_thread1 = threading.Timer(self.refresh_r,

            function=self.pic_to_text,

            kwargs={})

        self.new_thread1.daemon=True

        self.new_thread1.start()

        ##### if com port is open

        if serialPort.IsOpen():

            time_now=str(datetime.datetime.now()) # get time now

            ## convert image to tex

            text1=Img_to_Word.main(self.photo_img)

            message=time_now + " " + text1

            message += '\r\n'

            print(message)

            serialPort.Send(message)

            self.textbox.insert('1.0',message)

        else: ## if comport is closed

            self.textbox.insert('1.0', "Not sent - COM port is closed\r\n")

    ### used for snapshot button to extract text from image at a single moment

    def snapshot(self):

        time_now=str(datetime.datetime.now())

        #### image to text

        text1=Img_to_Word.main(self.photo_img)

        message=time_now+ " " + text1 #+'\r\n'

        ### used to save images

        #img =cv2.cvtColor(self.photo_img,cv2.COLOR_RGB2GRAY)

        #ne=time_now.split(".")

        #wr=ne[1]+".jpg"

        #cv2.imwrite(wr,img)

        message += '\r\n'

        print(message)

        if serialPort.IsOpen(): # if comport is open send message

            serialPort.Send(message)

            self.textbox.insert('1.0',message)

        else:# closed comport: write message but will not send

            self.textbox.insert('1.0',("WARNING closed comport, message not sent:   " + message))

    # Used to udate video in Tinker

    def update_Image(self):

        # Get a frame from the video source

        ret, frame = self.vid.get_frame()

        ### Get image

        if ret:

            self.photo_img=frame

            self.photo = PIL.ImageTk.PhotoImage(image = PIL.Image.fromarray(frame))

            self.canvas.create_image(0, 0, image = self.photo, anchor = tkinter.NW)

        #### Add thread to video queue

        try:

            res = self.thread_queue.get(0)

            self.text_label.config(text=res)

            self.window.after(100, self.update_Image)

        except queue.Empty:

            self.window.after(100, self.update_Image)

    ### clear the text box

    def ClearDataCommand(self):

        self.textbox.delete("1.0",tkinter.END)

    # # serial data callback function

    def OnReceiveSerialData(self,message):

        str_message = message.decode("utf-8")

        self.textbox.insert('1.0', str_message)

    # Register the callback above with the serial port object

    serialPort.RegisterReceiveCallback(OnReceiveSerialData)

    ## open the serial comport

    def OpenCommand(self):

        if self.button_openclose.cget("text") == 'Open COM Port':

            comport = self.comport_edit.get()

            baudrate = self.baudrate_edit.get()

            serialPort.Open(comport,baudrate)

            self.button_openclose.config(text='Close COM Port')

            self.textbox.insert('1.0', "COM Port Opened\r\n")

        elif self.button_openclose.cget("text") == 'Close COM Port':

            serialPort.Close()

            self.button_openclose.config(text='Open COM Port')

            self.textbox.insert('1.0',"COM Port Closed\r\n")

    ######## Change the refresh rate

    def Refresh_Rate(self):

        plholder=self.senddata_edit.get()

        #### tends to crash if refresh rate is to fast

        if int(plholder)>4.99:

            self.textbox.insert('1.0',("Refresh rate changed: "+plholder+ "\r\n"))

            self.refresh_r = int(plholder)

        else:

            self.textbox.insert('1.0',("Refresh rate WAS NOT changed please choose rate greater then 5s. \r\n"))

####### Create the video capture

class MyVideoCapture:

    def __init__(self, video_source=0):

        # Open the video source

        self.vid = cv2.VideoCapture(video_source)

        if not self.vid.isOpened():

            raise ValueError("Unable to open video source", video_source)

        # Get video source width and height

        self.width = self.vid.get(cv2.CAP_PROP_FRAME_WIDTH)

        self.height = self.vid.get(cv2.CAP_PROP_FRAME_HEIGHT)

    def get_frame(self):

        if self.vid.isOpened():

            ret, frame = self.vid.read()

            if ret:

                # Return a boolean success flag and the current frame converted to BGR

                return (ret, cv2.cvtColor(frame, cv2.COLOR_BGR2RGB))

            else:

                return (ret, None)

        else:

            return (None, None)

    # Release the video source when the object is destroyed

    def __del__(self):

        if self.vid.isOpened():

            self.vid.release()

#### multi threading

class ThreadedTask(threading.Thread):

    def __init__(self, queue):

        threading.Thread.__init__(self)

        self.thread_queue = queue

    def run(self):

        time.sleep(5)  # Simulate long running process

        self.thread_queue.put("Task finished")

### initialize tinker

root=tkinter.Tk()

App(root, "Tkinter and OpenCV")

### run system

root.mainloop()

sys.exit()

root.destroy()

Appendix A.2. Img_to_Word.py

import cv2

import numpy as np

import pytesseract

import imutils

import re

##### From the given a word and a number find the dose information

# because of the OCR nature we have two list one number (is just the tessaract OCR system looking only for numbers)

# word (is tessaract OCR looking for numbers and letters)

def order_output(word,number):

    ### create a list from all words and numbers

    words=word.upper().split("\n")

    numbers=number.split("\n")

    #print(words)

    #print(numbers)

    output="error" # final output of the data

    agent="" # agent name used

    regex = r'[0-9]' # sort for numbers

    numberlist=[] #list of numbers

    wordlist=[] #list of words

    nprev="" #placeholder previous nuber found

    nextnu=False # next string is a number?

    #### Go through the numbers for dose amount

    for n in numbers:

        if (re.match("_", n)!=None):#Blank found

            nprev=None

            continue

        if (re.match(regex, n)==None) or (len(n)<1):#no numbers found

            continue

        if nextnu==True:# string is a number

            numberlist.append(n+" " + nprev)#append it to the number list

            nextnu=False

        if ":" in n:# is it two number seprated by a :

            if n.rfind(":")<4:

                if nprev!=None:

                    numberlist.append(nprev+" " + n)

                else:

                    nextnu=True

        nprev=n

    numb2=[]

    pastele=''

    ###### go through the list of words

    for ele in words:

        if len(ele)<1:#is the word a blank ie. <1

            pastele=ele

            continue

        elif ele in pastele == True: # OCR will some times treat non-letter as letters they often are repeated

            pastele=ele

            continue

        pastele=ele

        if re.findall("[0-9]",ele)!=[]:# does the element have a number

            if re.findall("[a-zA-Z]",ele)!=[]: #does it have a letter

                non=1

            else: # the element maybe a number just not found

                numb2.append(ele)

        # dose thee element slightly match the desired values

        if "FENT" in ele or "TENT" in ele or "NTAN" in ele: # does the element match one of these then we assume

            if agent != "fentaNYL 1000: ": # is the past agent being repeated

                agent="fentaNYL 1000: "

                wordlist.append(agent)#add to the agent list

                numb2.append("dose_here")#add the dose here

        elif "SODI" in ele or "SODJ" in ele or "SADI" in ele:

            if agent != "Sodium Cl 3%: ":

                agent="Sodium Cl 3%: "

                wordlist.append(agent)

                numb2.append("dose_here")

    #print(words)

    #print(wordlist)

    #print(numb2)

    #print(numberlist)

    ### compare the list of words and number to create the dosing information

    if len(numberlist)==len(wordlist):# if they are the same then we have only one dose amount and agent

        output=""

        for i in range(len(numberlist)):

            output+=(wordlist[i]+" "+numberlist[i]+" ")

        return output

    elif numb2.count("dose_here")==len(wordlist):#if we have a miss match replace dose here with actual amount

        stto=0

        output=""

        for i in range(len(wordlist)):

            stto= numb2.index("dose_here",stto)+1

            if numb2[stto-3] != "dose_here":# is volume recored or not

                output+=(wordlist[i]+" "+numb2[stto-3]+" ")

            else:

                output+=(wordlist[i]+" "+numb2[stto-2]+" ")

        return output

    else:# there is a missmatch between dose agent and amount

        output= ["Error miss match"]

        return output

#### resize the image

def resize_img(img, percent):

    width = int(img.shape[1] * percent / 100)

    height = int(img.shape[0] * percent / 100)

    dim = (width, height)

    # resize image

    imgout = cv2.resize(img, dim, interpolation = cv2.INTER_CUBIC)

    return imgout

### used to find borders of key image

def find_border_components(contours, ary):

    key_contours=[]

    img_h=ary.shape[0]

    img_w=ary.shape[1]

    for i, c in enumerate(contours):

        x,y,w,h = cv2.boundingRect(c)

        if (h*w)>(0.3*img_h*img_w):

            key_contours.append((x,y,w,h))

    if len(key_contours)==0:

        return

    key_contours.sort(key=lambda x:(x[2]*x[3]))

    #print(key_contours)

    borders=(key_contours[0][0],key_contours[0][1],key_contours[0][0]+key_contours[0][2]+key_contours[0][0],key_contours[0][1]+key_contours[0][3])

    return borders

#### main function takes an image and converts it to dosing information

def main(imgin):#input is a RGB array image

    img =cv2.cvtColor(imgin,cv2.COLOR_RGB2GRAY)# convert to greyscale

    #img=cv2.imread(imgin,0)

    edges = cv2.Canny(img,100,200) # find all the edges

    kernel = np.ones((2,2), dtype=np.uint8) # define a kernel size used to find the edges

    edges=cv2.dilate(edges, kernel, iterations=3) # find the images edges

    #cv2.imwrite("B.jpg",edges)

    contours, hierarchy = cv2.findContours(edges, cv2.RETR_TREE, cv2.CHAIN_APPROX_SIMPLE) #use edges to create contours

    borders = find_border_components(contours, edges) # Note the main disply must be atleast 50% of display window

    if borders!=None:

        img=img[borders[1]:borders[3],borders[0]:borders[2]]

    ### resize the image based on borders

    img = resize_img(img,400)

    #cv2.imwrite("C.jpg",img)

    ### black and white the image to get solid letters and numbers

    if np.median(img) < 200:

        img = 255-img

    img=cv2.adaptiveThreshold(img, 255, cv2.ADAPTIVE_THRESH_MEAN_C,cv2.THRESH_BINARY, 101, 23)

    #cv2.imwrite("D.jpg",img)

    ## Again find the edges and then reshape

    edges = cv2.Canny(img,100,200)

    kernel = np.ones((3,3), dtype=np.uint8)

    edges=cv2.dilate(edges, kernel, iterations=6)

    #cv2.imwrite("E.jpg",edges)

    #### check for image rotation

    contours, hierarchy = cv2.findContours(edges, cv2.RETR_TREE, cv2.CHAIN_APPROX_SIMPLE)

    height, width = img.shape

    h_len=height

    rect=((width/2,height/2),(height,width),0)

    for i, c in enumerate(contours):

        ((x,y), (h,w),rotation) = cv2.minAreaRect(c)

        if w>width*0.5:

            if h<h_len:

                rect=cv2.minAreaRect(c)

                h_len=h

    ((x,y), (h,w),rotation)=rect

    box = cv2.boxPoints(rect)

    box = np.int0(box)

    img2=cv2.cvtColor(img,cv2.COLOR_GRAY2RGB)

    img2=cv2.drawContours(img2,[box],0,(0,0,255),10)

    #print(rotation)

    ####  rotate image

    if rotation!=None and rotation<-45:

        img = imutils.rotate(img, angle=90+rotation)

    #### trim after rotation

    if borders!=None:

        if round(x-w/2)<60:

            img=img[round(height*0.05):round(height*0.9),60:round(x+w/2)]

        else:

            img=img[round(height*0.05):round(height*0.9),round(x-w/2):round(x+w/2)]

    #cv2.imwrite("F.jpg",img)

    ## use tesseract to assess the image

    d = pytesseract.image_to_data(img, output_type=pytesseract.Output.DICT)

    # from the tesseract image found object extract them and put them into a vertical image

    n_boxes = len(d['level'])

    maxw=np.max(d['width'])

    full=np.ones((1,round(maxw/2)+10))*255

    for i in range(n_boxes):

        (x, y, w, h) = (d['left'][i], d['top'][i], d['width'][i], d['height'][i])

        #cv2.rectangle(img4, (x, y), (x + w, y + h), (0, 255, 0), 2)

        if w<round(maxw/2):

            if h<200:

                bas=np.ones((h+20,round(maxw/2)+10))*255

                #print(h,w,maxw,x+w-x,w+10-10,round(maxw/2))

                crop_img = img[y:(y+h), x:(x+w)]

                bas[0:h, 10:(w+10)]=crop_img

                full=np.vstack((full,bas))

    img=full

    #cv2.imwrite("G.jpg",img)

    #### use OCR tesseract to find all words and numbers

    custom_config = r'-c tessedit_char_whitelist=0123456789.:_ --psm 11'

    final_num= (pytesseract.image_to_string(img, config=custom_config))

    custom_config = r'-c tessedit_char_blacklist=\"--psm 11'

    final_text= (pytesseract.image_to_string(img, config=custom_config))

    ###### sort the words and numbers from OCR

    worder= order_output(final_text,final_num)

    #print(worder)

    return worder

Appendix A.3. serial_rx_tx.py

import serial

import sys

import _thread

#### declaring a serial port

class SerialPort:

    def __init__(self):

        self.comportName = ""

        self.baud = 0

        self.timeout = None

        self.ReceiveCallback = None

        self.isopen = False

        self.receivedMessage = None

        self.serialport = serial.Serial()

    def __del__(self):

        try:

            if self.serialport.is_open():

                self.serialport.close()

        except:

            print("Error, closing COM port: ", sys.exc_info()[0] )

    def RegisterReceiveCallback(self,aReceiveCallback):

        self.ReceiveCallback = aReceiveCallback

        try:

            _thread.start_new_thread(self.SerialReadlineThread, ())

        except:

            print("Error, starting thread read: ", sys.exc_info()[0])

    def SerialReadlineThread(self):

        while True:

            try:

                if self.isopen:

                    self.receivedMessage = self.serialport.readline()

                    if self.receivedMessage != "":

                        self.ReceiveCallback(self.receivedMessage)

            except:

                print("Error reading COM port: ", sys.exc_info()[0])

    def IsOpen(self):

        return self.isopen

    def Open(self,portname,baudrate):

        if not self.isopen:

            self.serialport.port = portname

            self.serialport.baudrate = baudrate

            try:

                self.serialport.open()

                self.isopen = True

            except:

                print("Error, opening COM port: ", sys.exc_info()[0])

    def Close(self):

        if self.isopen:

            try:

                self.serialport.close()

                self.isopen = False

            except:

                print("Close error, closing COM port: ", sys.exc_info()[0])

    def Send(self,message):

        if self.isopen:

            try:

                # Ensure that the end of the message has both \r and \n, not just one or the other

                newmessage = message.strip()

                newmessage += '\r\n'

                self.serialport.write(newmessage.encode('utf-8'))

            except:

                print("Error, sending message: ", sys.exc_info()[0] )

            else:

                return True

        else:

            return False
